# Multi-Center Comparison of Two Self-Expanding Transcatheter Heart Valves: A Propensity Matched Analysis

**DOI:** 10.3390/jcm11144228

**Published:** 2022-07-21

**Authors:** Johannes Blumenstein, Clemens Eckel, Oliver Husser, Won-Keun Kim, Matthias Renker, Yeong-Hoon Choi, Christian W. Hamm, Hani Al-Terki, Dagmar Sötemann, Leon Körbi, Vedat Tiyerili, Christina Grothusen, Luise Gaede, Guido Dohmen, Helge Möllmann

**Affiliations:** 1 Department of Internal Medicine I, St.-Johannes-Hospital, 44137 Dortmund, Germany; clemens.eckel@joho-dortmund.de (C.E.); oliver.husser@gmail.com (O.H.); hani.alterki.1990@gmail.com (H.A.-T.); dagmar.soetemann@joho-dortmund.de (D.S.); leon.koerbi@joho-dortmund.de (L.K.); vedat.tiyerili@joho-dortmund.de (V.T.); christina.grothusen@joho-dortmund.de (C.G.); helge.moellmann@joho-dortmund.de (H.M.); 2 Medical School, Carl von Ossietzky University, 26111 Oldenburg, Germany; 3 Department of Cardiology, Kerckhoff Heart Center, 61231 Bad Nauheim, Germany; w.kim@kerckhoff-klinik.de (W.-K.K.); m.renker@kerckhoff-klinik.de (M.R.); christian.hamm@innere.med-giessen.de (C.W.H.); 4 Department of Cardiac Surgery, Kerckhoff Heart Center, 61231 Bad Nauheim, Germany; y.choi@kerckhoff-klinik.de; 5 Department of Cardiology, Justus-Liebig University of Giessen, 35390 Giessen, Germany; 6 Department of Medicine 2-Cardiology and Angiology, Friedrich-Alexander University, 91054 Erlangen, Germany; luise.gaede@uk-erlangen.de; 7 Department of Cardiothoracic Surgery, St.-Johannes-Hospital, 11137 Dortmund, Germany; guido.dohmen@joho-dortmund.de

**Keywords:** TAVR, transfemoral, self-expanding, balloon-expanding, aortic stenosis

## Abstract

Background: During the last years, several transcatheter aortic heart valves entered the clinical market and are commercially available. The prostheses differ regarding several technical and functional aspects. However, little is known regarding head-to-head comparative data of the ACURATE neo and the PORTICO valve prostheses. Objectives: The aim of this study was to compare two self-expanding transcatheter aortic heart valves (THV), the ACURATE neo and the PORTICO, with regard to in-hospital and 30-day outcomes, as well as early device failures. Methods: A total of 1591 consecutive patients with severe native aortic valve stenosis from two centers were included in the analyses and matched by 1:1 nearest neighbor matching to identify one patient treated with PORTICO (*n* = 344) for each patient treated with ACURATE neo (*n* = 344). Results: In-hospital complications were comparable between both valves, including any kind of stroke (ACURATE neo = 3.5% vs. PORTICO = 3.8%; *p* = 1.0), major vascular complications (ACURATE neo = 4.5% vs. PORTICO = 5.4%; *p* = 0.99) or life-threatening bleeding (ACURATE neo = 1% vs. PORTICO = 2%; *p* = 0.68). The rate of device failure defined by the VARC-2 criteria were comparable, including elevated gradients and moderate-to-severe paravalvular leakage (ACURATE neo = 7.3% vs. PORTICO = 7.6%; *p* = 1.0). However, the need for permanent pacemaker implantation (PPI) was significantly more frequent after the use of PORTICO THV (9.5% vs. 18.7%; *p* = 0.002). Conclusions: In this two-center case-matched comparison, short-term clinical and hemodynamic outcomes showed comparable results between PORTICO and ACURATE neo prostheses. However, PORTICO was associated with a significant higher incidence of PPI.

## 1. Introduction

Transcatheter aortic valve replacement (TAVR) became a highly standardized procedure for the treatment of high, intermediate and even low-risk patients suffering from severe aortic stenosis [1,2,3,4,5,6,7]. While, in the beginning, only two different devices were available, nowadays, several different devices have entered the clinical market.

Both valve types, balloon-expandable and self-expanding, have revealed high clinical and procedural success rates at the first glance. However, the devices differ not only in regard to the stent and device design but also with respect to the mechanism of deployment, sizing range, hemodynamic performance and risk of atrioventricular conductance disturbances (CDs). Initially, the decision between the valve models was mainly driven by local expertise. With a growing number of procedures, comparative data from randomized, multicenter trials could demonstrate the effect of different valve prostheses properties on the clinical outcomes [8,9,10,11,12,13,14].

The ACURATE neo (Boston Scientific, Marlborough, MA, USA) and the PORTICO (Abbott Structural Heart, St. Paul, MN, USA) transcatheter heart valves are self-expanding TAVR prostheses associated with favorable outcomes in nonrandomized studies [15,16,17].

However, a direct head-to-head comparison of both valve types is lacking.

Therefore, the aim of this two-center, propensity-matched analysis was a direct comparison of the ACURATE neo vs. the PORTICO valve in terms of device success and early (30-day) outcomes according to the criteria of the Valve Academic Research Consortium.

## 2. Materials and Methods

### 2.1. Patient Population

A total of 1591 consecutive patients with severe and symptomatic stenosis of the native aortic valve in 2 centers in Germany underwent transfemoral TAVR using either ACURATE neo (*n* = 1247) or PORTICO (*n* = 344). All implanting interventionalists of both centers were highly experienced, with both valve types and both centers providing a balanced rate of patients, which were treated using a comparable standard (Bad Nauheim: ACURATE = 625; PORTICO = 145/Dortmund: ACURATE = 622; PORTICO = 199). All patients were discussed by a local interdisciplinary heart team and were selected for a transfemoral aortic valve implantation according to the existing guidelines [18,19]. Final decision of the valve type and size was left at the discretion of the implanting physician according to the suggestions of the manufacturer. All patients gave written consent for the procedures.

### 2.2. Multislice Computed Tomography Data Analysis

Multislice computed tomography (MSCT) was performed as part of the standard protocol for preprocedural screening. The aortic annulus measurements were evaluated after reconstruction on several levels, according to the guidelines of the Society of Cardiovascular Computed Tomography [20]. The area, perimeter and the maximum and minimum diameters of the virtual aortic annulus were calculated by direct planimetry and length measurements. The eccentricity of the annulus (incl. index) was also calculated with a limit value for an eccentric ring for an eccentricity index > 0.25 [8]. The calcification of the aortic valve was evaluated visually, categorized as mild, moderate or severe.

### 2.3. Device Description

ACURATE neo is a supra-annular valve that is available in 3 sizes (small, medium and large). The technical features have already been described [21]. The device consists of a self-expanding nitinol frame with a porcine pericardial leaflets valve in a supra-annular position and a pericardial sealing skirt on the outer and inner surfaces of the stent body. ACURATE neo is delivered transfemorally using the ACURATE neo/TF delivery system compatible with a 15-F–18-F catheter sheath (internal diameter). Recently, a new 14-F sheath expanding to 21-F during the passing of the prepared prosthesis, the iSleeve, became available and was also used in a study cohort.

PORTICO is a resheathable, intra-annular valve that is available in 4 sizes (23, 25, 27 and 29 mm). It consists of a self-expanding nitinol frame with bovine pericardial leaflets and a porcine pericardial sealing cuff. The valve is delivered using the flexible PORTICO Delivery system, which has an 18-F outer diameter for the small valves (23 and 25 mm) and a 19-F outer diameter for the larger valves (27 and 29 mm). The system has the ability to retrieve and reposition the PORTICO valve until 80% deployment. Further technical features have already been described in detail [17]. Recently, a novel 15-F sheathless delivery system (Flexnav) became available and was also used in the present cohort.

The technical features of both THVs, their sheath dimensions and sizing recommendations are summarized in Online Table S1.

### 2.4. Definition of Endpoints and Follow-Up

The primary endpoints were in-hospital and 30-day mortality. The secondary outcome measures were device success, including technical success and the early safety combined endpoint at 30 days, according to the VARC-2 criteria. Follow-up to 30 days was prospectively collected at each of the participating sites in the outpatient clinic, contacting the primary care physician or by direct contact with the patient. Transthoracic echocardiography was performed at the baseline and before discharge.

### 2.5. Statistical Analysis

**Figure 1:**
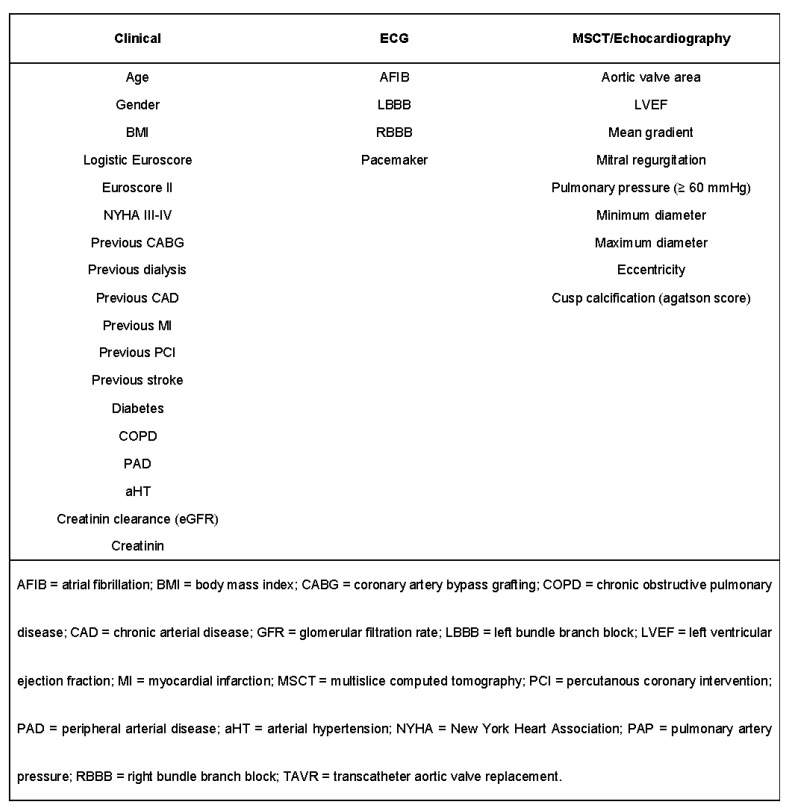
Variables used for the propensity matching.

The categorical variables were expressed as counts (percentages), and continuous variables were expressed as the mean (SD) or the median (interquartile range) and compared using the Student’s *t*-test or Mann–Whitney *U* test, respectively. To reduce the imbalance in the patient baseline characteristics and the effect of a potential selection bias on both endpoints for comparing ACURATE neo with PORTICO, propensity matching was performed using R Studio version 1.2.5042 (The R Foundation for Statistical Computing, Vienna, Austria). A 1-to-1 nearest neighbor matching was used to identify one control case treated with PORTICO (*n* = 344) for each case treated with ACURATE neo (*n* = 344). The baseline clinical history, electrocardiogram and MSCT characteristics with known impacts or showing significant (*p* < 0.05) univariate differences between both groups were included in the matching algorithm (Figure 1).

The occurrence of VARC-2-defined in-hospital complications were determined for the total and the matched populations. A logistic regression analysis was used to determine the predictors of device failure. A Cox proportional hazard analysis, as well as the Kaplan–Meier method, were used to calculate the mortality after 30 days and were indicated by hazard ratios (HRs) and 95% confidence intervals (CIs). The data for the follow-up analysis after 30 days were available for 92.6% (1474 out of 1591) of the entire population and for 91.7% (631 out of 688) of the matched population.

All analyses were carried out in the matched population, as well as the subgroups of the patients with device failures and with indications of new pacemaker implantations. A 2-sided *p*-value of <0.05 was considered statistically significant for all analyses.

## 3. Results

### 3.1. Baseline Characteristics and Propensity Matching

**Figure 2:**
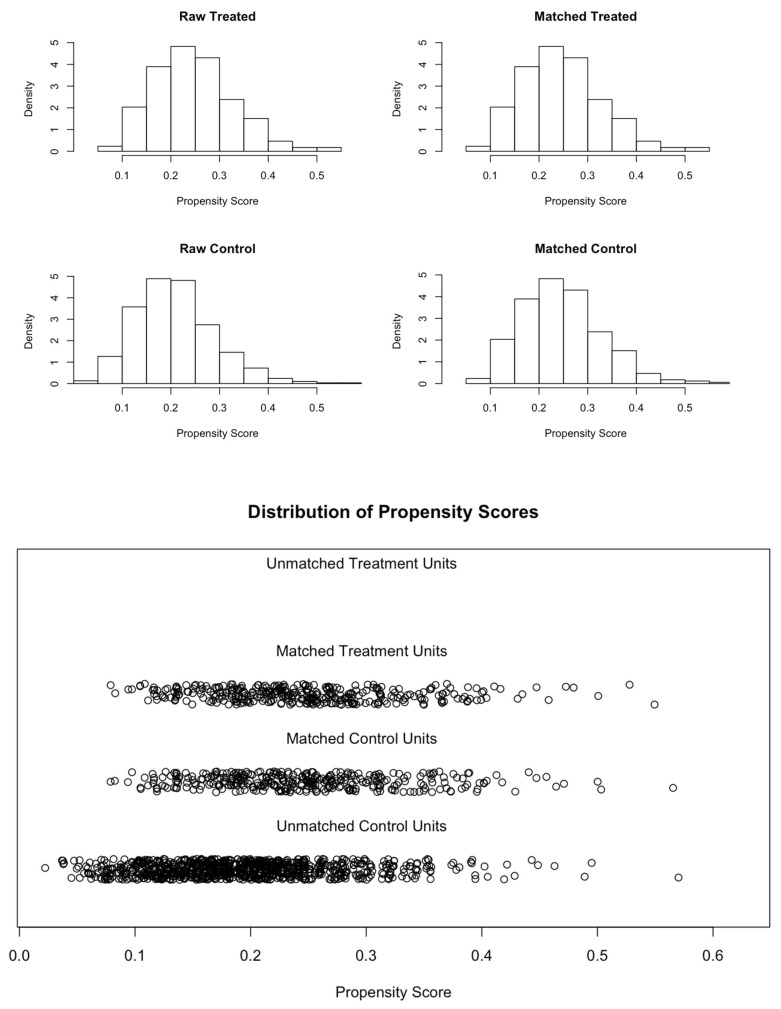
Propensity scores in the treated and control groups.

The baseline characteristics of the entire cohort, as well as of the matched cohort, are displayed in Table 1. In comparison to the patients treated with PORTICO, the ACURATE neo recipients were significantly younger, had a tendency for a better left ventricular ejection fraction (62% vs. 60%; *p* = 0.057), lower mean transaortic gradient (41 mmHg vs. 43 mmHg; *p* = 0.006) and a higher prevalence of right bundle branch blocks (10.7% vs. 5.0%; *p* = 0.002) in the entire population. Patients treated with ACURATE neo THV had a significantly lower percentage of heavily calcified aortic valve morphology than patients treated with the PORTICO device. In the matched population, the baseline characteristics were similar between the two groups (Table 1). The quality of the propensity matching is visualized in Figure 2.

### 3.2. Procedural Data and In-Hospital Outcome of the Matched Cohort

The procedural details and in-hospital complications are summarized in Table 2 (see Online Table S2 for the analysis with entire population). The majority of the matched population received TAVR under conscious sedation (PORTICO 89.5% vs. ACURATE neo 87.2%; *p* = 0.405). Pre-dilatation was similar (PORTICO 79.9% vs. 78.5%; *p* = 0.707), while post-dilation was significantly more common with ACURATE neo (18.6% vs. 32.0%; *p* < 0.001). The small, medium and large sizes of ACURATE neo were used in 14.2%, 43.0% and 42.7% of the cases, while 23, 25, 27 and 29 mm of PORTICO were used in 1.7%, 27.0%, 35.8% and 35.5%. The total fluoroscopy time was significantly shorter for ACURATE neo implantation (10.3 min vs. 12.5 min; *p* < 0.001), and less contrast agent was used compared to PORTICO (100 mL vs. 120 mL; *p* < 0.001).

There were no differences between the matched populations regarding incidences of stroke, severe vascular complications, life-threatening bleeding, kidney failure or myocardial infarction (Table 2). Patients treated with PORTICO had a higher rate of new permanent pacemaker implantations (PPI) (9.5% vs. 18.7%; *p* = 0.002). PORTICO was an independent predictor of pacemaker implantations (OR 1.78; 95% CI 1.25–2.53; *p* = 0.001). Overall, the patients in need of new PPI more frequently had preexisting RBBB (new PPI 39.7% vs. no PPI 10.0%, *p* < 0.001). The length of stay was comparable between both groups. The in-hospital all-cause mortality was 2.9% for ACURATE neo and 2.9% for PORTICO, without significant differences.

### 3.3. Device Failure According to VARC-2

Device failure for PORTICO and ACURATE neo (see Online Table S3 for the analysis of the entire population) was comparable (ACURATE neo 7.3% vs. PORTICO 7.6% *p* = 1.000). The mean transvalvular gradients decreased post-procedurally with both THVs without significant differences. Moderate-to-severe paravalvular leakage (PVL) was similar for both devices (ACURATE neo 4.8% vs. PORTICO 3.5%, *p* = 0.546).

Urgent conversion to sternotomy was rare in both groups (ACURATE neo 0.6% vs. PORTICO 1.5%; *p* = 0.451). Conversion followed coronary impairment (one), THV embolization (five), pericardial effusion (five) and severe mitral regurgitation due to wire perforation (one) (see Online Table S4). The procedural mortality was 2.0% for ACURATE neo and 0.5% for PORTICO (*p* = 0.623). The details for device failure are given in Table 3 and Table 4.

### 3.4. 30-Day Follow-Up

**Figure 3:**
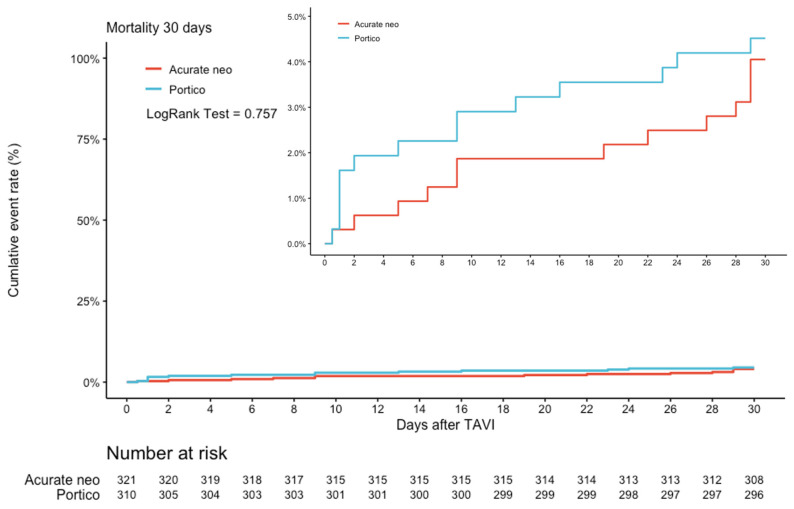
Kaplan–Meier cumulative event rate for the 30-day mortality. Cumulative event rate after 30 days for mortality in ACURATE neo vs. PORTICO (HR 1.13; 95% CI 0.53–2.40, *p* = 0.757).

There were no significant differences regarding mortality after 30 days (3.8% vs. 4.1%, RR 1.13, 95% CI 0.53–2.40, *p* = 0.757) in the matched population (Figure 3).

## 4. Discussion

As TAVR has become a highly standardized procedure not only in regard to the preprocedural diagnostics but also the periprocedural workflow, the overall procedural success rate is very high.

During the last years, several TAVR devices, including specific iterations to address typical complications that are known to influence the outcome, entered the clinical market.

While, initially, valve selection was based on the individual experience of the interventionalist, with growing numbers of procedures and increasing experience, comparative studies including different TAVR devices have been published. In the SCOPE I trial, ACURATE neo did not meet noninferiority compared to the balloon-expandable SAPIEN 3 device in terms of early safety (30 days) and clinical efficacy outcomes [11]. This was mainly driven by differences in the severity of the PVL and the frequency of stage 2 or 3 acute kidney injury. Interestingly, in contrast to prior nonrandomized trials, the rates of new pacemaker implantations were comparable between ACURATE neo and SAPIEN 3. Comparably to prior results, echocardiography revealed smaller gradients and larger effective orifice areas after ACURATE neo prostheses [22]. At 1 year, no differences in the clinical outcomes were observed in the randomized comparison. The rate of PVL remained higher and the transprosthetic mean gradients lower in ACURATE neo recipients from 30 days to the 1-year follow-up [23]. In addition to this, the SCOPE II trial of ACURATE neo did not meet noninferiority compared to the self-expanding CoreValve Evolute device in terms of all-cause death or stroke at 30 days and 1 year. However, ACURATE neo was associated with a lower incidence of permanent pacemakers. Nevertheless, a secondary analysis revealed a higher incidence of moderate or severe regurgitation at 30 days and cardiac death at 30 days and 1 year after ACURATE neo compared to CoreValve Evolute [14].

The randomized PORTICO IDE trial compared the PORTICO valve with different commercial valves. It revealed a higher rate of safety endpoint events, including mortality, of the PORTICO valve than the commercial valves at 30 days [12]. In addition, the rates of permanent pacemaker implantation and moderate or greater PVL were higher in the PORTICO valve group than in the commercial valve group (Edwards SAPIEN 3, CoreValve Evolute R/Pro). A post hoc comparison showed increased mortality at 2 years in the PORTICO group compared with the SAPIEN 3 valve and a similar mortality in Evolut R and Evolut PRO valves. However, in a post-hoc analysis, superiority was not reached for the Edwards SAPIEN or the Medtronic CoreValve prostheses.

To the best of our knowledge, this is the first two-center, propensity-matched comparison of the ACURATE neo and the PORTICO TAVR valve prostheses.

Consistent with prior publications, in our analysis, both valves showed high procedural success rates (92.7% for the ACURATE neo and 92.4% for the PORTICO valve) [12,22].

As a main finding of our trial, no significant differences could be observed in regard to procedural success and intrahospital and 30-day mortality.

### 4.1. Echocardiographic Data

Residual PVL after TAVR is generally associated with adverse outcomes [11,12,24]. In our trial, both valves demonstrated low rates of more than mild PVL, which was comparable to the post-market follow-up data [16,25,26]. Due to the important impact of residual regurgitation, newer generations of both valves have an additional skirt to further reduce the incidence of PVL.

Despite the intra-annular design of the PORTICO valve, the hemodynamics, including the aortic valve area and aortic valve gradients at discharge, were comparable with the supra-annular ACURATE neo valve. This is remarkable, since the supra-annular design was believed to be associated with improved hemodynamics compared to intra-annular valves [8]. However, this effect may be more meaningful in patients with small annuli, which were underrepresented in our PORTICO cohort at only 1.7%.

### 4.2. Access-Related Complications and Major Bleeding Events

Access-related complications and major bleedings events are known to be strongly related to adverse outcomes [2,5,27,28]. With the development of next-generation transfemoral THV, delivery systems have been optimized to minimize vascular complications and bleeding [29].

Major vascular complications and major bleeding have been reported in about 8% with ACURATE neo and in about 6% with PORTICO [11,12].

In our cohort, major bleeding events were rare, and no differences between ACURATE neo and PORTICO for major vascular complications and for life-threatening bleeding were observed.

However, to further reduce this important complication, recently, a new 14-F introducer sheath, expanding to 21-F during passing of the prepared prosthesis, for the implantation of the ACURATE neo and a new generation introducer sheath for the PORTICO valve, which can be inserted sheathlessly, became available. Both devices were only used in a relatively small proportion in the study cohort; therefore, an intercomparison would not have been meaningful.

### 4.3. Permanent Pacemaker Implantation

A main finding of the present study was a significantly higher incidence of PPI in the PORTICO group. Differences in the radial force and the stent design, including different implantation techniques (ACUARET neo: top-down vs. PORTICO: bottom-up deployment) with potentially less interference with the conduction system, may be an important explanation for this result. In line with prior data, preexisting RBBB was an independent predictor for the need of PPI in the overall cohort [30]. Furthermore, the implantation depth has an important impact on the incidences of conduction disturbances [31]. Therefore, a high prosthesis position should always be attempted, which might be a reason for the relatively high number of embolizations in the portico arm. In this context, the cusp overlay technique gave some important impact recently [32,33]. Even though PPI may not have any impact on short-term outcomes, potentially detrimental effects on mortality may become apparent in the long-term [34,35,36]. It has to be taken into account that the data were collected mainly from elderly patients with other comorbidities, influencing the life expectancy. Therefore, the real issue of long-term right ventricular pacing demonstrated in other settings may not be readily apparent in the sicker TAVR population. New PPI after TAVR using earlier generation ranged between 5% and 12% for balloon-expandable and 28% in self-expanding devices [37]. In contrast to a significant reduction of other typical TAVR complications, such as PVL, the available evidence to date does not support a dramatic reduction in the PPM rates since the arrival of newer-generation devices [38]. As the strategies and indications for permanent pacemaker implantations differ significantly between centers, recently, the first recommendations of the management of conduction disturbances associated with TAVR were published [39].

### 4.4. Limitation

The lack of randomization is an obvious limitation of the present study; however, the main strength of the present study is the relatively high number of patients and the use of a case-matching procedure using a propensity score to achieve comparable groups of patients with both valves to be included in the analysis. This is a real-world study and reflects the current clinical practices in two high-volume centers performing TAVR using both systems for many years in Germany. All implanting interventionalists were highly experienced with both devices, and both centers included a balanced rate of patients. Even though the patients were treated using comparable procedural standards, a minor bias could not be excluded.

Generally, it has to be taken into account that both valve prostheses have a known learning curve, which might have an effect at less experienced centers.

## 5. Conclusions

In this two-center case-matched comparison, the short-term clinical and hemodynamic outcomes showed comparable results between PORTICO and ACURATE neo prostheses. However, PORTICO was associated with a significantly higher incidence of PPI.

## Figures and Tables

**Table 1:** Baseline characteristics of the patients treated with ACURATE neo and of the entire population and the matched population treated with PORTICO.

		Entire Population		Matched Population (1:1)	
	PORTICO	ACURATE Neo	*p*-Value	ACURATE Neo	*p*-Value
	(*n* = 344)	(*n* = 1247)		(*n* = 344)	
**Clinical characteristics**					
Age	83.0 (79.7–86.0)	82.0 (78.6–85.0)	0.003	82.8 (79.6–86.0)	0.863
Gender (male):	138 (40.1%)	484 (38.8%)	0.707	149 (43.3%)	0.439
BMI (kg/m^2^)	26.7 (24.2–30.7)	26.6 (23.9–30.4)	0.533	26.9 (24.0–30.9)	0.905
Log Euroscore (%)	17.4 (11.2–26.8)	17.5 (11.2–26.4)	0.841	17.4 (11.6–26.8)	0.911
Euroscore II (%)	4.2 (2.7–7.0)	4.1 (2.6–6.8)	0.344	4.2 (2.6–7.2)	0.731
NYHA (III/IV)	290 (84.3%)	1008 (80.8%)	0.164	298 (86.6%)	0.449
COPD	70 (20.3%)	241 (19.3%)	0.729	72 (20.9%)	0.925
Hypertension	308 (89.5%)	1121 (89.9%)	0.924	313 (91.0%)	0.607
Diabetes	120 (34.9%)	400 (32.1%)	0.359	130 (37.8%)	0.476
eGFR (mL/min)	54.0 (41.8–74.2)	59.0 (43.0–78.0)	0.059	59.0 (42.0–76.2)	0.423
Creatinine (mg/dL)	1.0 (0.8–1.3)	1.0 (0.8–1.3)	0.346	1.0 (0.8–1.3)	0.971
Dialysis	6 (1.7%)	32 (2.6%)	0.494	4 (1.2%)	0.750
PAD	48 (14.0%)	173 (13.9%)	1.000	45 (13.1%)	0.824
Previous Stroke	31 (9.0%)	168 (13.5%)	0.034	27 (7.8%)	0.681
CAD	230 (66.9%)	821 (65.8%)	0.772	227 (66.0%)	0.872
Previous MI	37 (10.8%)	142 (11.4%)	0.817	33 (9.6%)	0.705
Previous PCI	129 (37.5%)	471 (37.8%)	0.977	124 (36.0%)	0.752
Previous CABG	31 (9.0%)	125 (10.0%)	0.648	31 (9.0%)	1.000
**Echocardiographic characteristics**					
LVEF (%)	60.0 (52.0–65.0)	62.0 (53.0–65.0)	0.057	60.0 (50.0–65.0)	0.474
Pmean (mmHg)	43.0 (35.0–50.0)	41.0 (31.0–49.0)	0.006	41.0 (32.0–51.0)	0.405
MR (≥Grade II)	5 (1.5%)	11 (0.9%)	0.361	5 (1.5%)	1.000
TR (≥Grade II)	5 (1.5%)	17 (1.4%)	0.800	4 (1.2%)	1.000
sPAP (≥60 mmHg)	25 (8.9%)	94 (9.0%)	1.000	32 (9.3%)	1.000
**Electrocardiographic characteristics**					
Atrial fibrillation	135 (39.2%)	479 (38.4%)	0.827	129 (37.5%)	0.695
LBBB	34 (10.0%)	109 (8.8%)	0.568	36 (10.5%)	0.900
RBBB	17 (5.0%)	133 (10.7%)	0.002	14 (4.1%)	0.713
Pacemaker	66 (19.2%)	147 (11.8%)	0.001	49 (14.2%)	0.102
**MSCT data**					
Prosthesis area (cm^2^)	4.3 (4.0–4.9)	4.5 (4.0–4.9)	0.435	4.6 (4.1–4.9)	0.064
Minimum diameter (mm)	20.7 (19.2–22.0)	20.3 (19.0–21.8)	0.007	20.9 (19.6–22.0)	0.197
Maximum diameter (mm)	26.9 (25.4–28.2)	26.7 (25.1–28.0)	0.131	27.0 (25.7–28.4)	0.431
Distance to LCA (mm)	13.0 (11.6–15.0)	13.1 (11.1–15.2)	0.966	13.1 (11.9–15.4)	0.321
Distance to RCA (mm)	17.0 (15.0–19.0)	17.0 (15.0–19.0)	0.422	17.1 (15.4–19.0)	0.040
Eccentricity	0.2 (0.2–0.3)	0.2 (0.2–0.3)	0.057	0.2 (0.2–0.3)	0.630
Calcification (AU)	2366.0 (1661.0–3371.0)	2197.5 (1400.5–3187.2)	0.015	2512.0 (1641.0–3435.0)	0.743

Values are the mean ± SD, *n* (%) or median (interquartile range). CABG = coronary artery bypass grafting; COPD = chronic obstructive pulmonary disease; CAD = coronary artery disease; MI = myocardial infarction; PAD = peripheral artery disease; eGFR = creatinine clearance; LBBB = complete left bundle branch block; LV = left ventricular; EF = Ejection fraction; MR = Mitral Regurgitation, TR = tricuspid regurgitation, sPAP = systolic pulmonary arterial pressure; MSCT = multislice computed tomography; NYHA = New York Heart Association functional class; PCI = percutaneous coronary intervention; RBBB = complete right bundle branch block, AU = Angaston units.

**Table 2:** Procedural characteristics and in-hospital complications of the patients treated with PORTICO and of the entire population and the matched population treated with ACURATE neo.

		Matched Population (1:1)	
	PORTICO	ACURATE Neo	*p*-Value
	(*n* = 344)	(*n* = 344)	
**Procedural data**			
Conscious sedation	308 (89.5%)	300 (87.2%)	0.405
THV Size			<0.001
23	6 (1.7%)	49 (14.2%)	
25	93 (27.0%)	148 (43.0%)	
27	123 (35.8%)	147 (42.7%)	
29	122 (35.5%)	0 (0.0%)	
Pre-dilatation	275 (79.9%)	270 (78.5%)	0.707
Post-dilatation	64 (18.6%)	110 (32.0%)	<0.001
Cerebral protection	2 (0.6%)	7 (2.0%)	0.177
Procedural time (min)	52.0 (40.0–69.0)	56.0 (38.0–72.2)	0.853
Contrast (mL)	120.0 (100.0–160.0)	100.0 (80.0–130.0)	<0.001
Fluoroscopy dose (Gy)	1466.5 (29.1–3463.8)	1391.0 (27.1–3635.5)	0.868
Fluoroscopy time (s)	12.5 (9.2–17.1)	10.3 (8.0–14.6)	<0.001
**Echocardiographic characteristics**			
Postprocedural mean gradient (mmHg)	8.0 (6.0–10.0)	8.0 (6.0–10.0)	0.982
Postprocedural max gradient (mmHg)	13.0 (10.0–18.0)	14.0 [11.0;18.0]	0.856
**Clinical events**			
Major stroke/minor stroke/TIA	13 (3.8%)	12 (3.5%)	1.000
Major vascular complications	9 (4.5%)	11 (5.4%)	0.854
Life-threatening bleeding (VARC)	2 (1.0%)	4 (2.0%)	0.685
Renal failure (AKIN 2/3)	12 (3.5%)	13 (3.8%)	0.994
Coronary artery obstruction with PCI	0 (0.0%)	3 (1.5%)	0.248
Myocardial infarction	6 (3.0%)	8 (3.9%)	0.815
Permanent pacemaker implantation ^1^	52 (18.7%)	28 (9.5%)	0.002
Days in hospital	7.0 (6.0–10.0)	7.5 (6.0–11.0)	0.965
Days in the intensive care unit	2.0 (1.0–3.0)	2.0 (1.0–3.0)	0.825
In-hospital mortality	10 (2.9%)	10 (2.9%)	1.000

Values are the mean ± SD, *n* (%) or median (interquartile range). ^1^ Excluding patients with pacemakers at the baseline (*n* = 115 in the matched population). VARC = Valve Academic Research Consortium, AKIN = Acute Kidney Injury Network, TIA = transitory ischemic attack.

**Table 3:** Device failure for patients treated with PORTICO and ACURATE neo of the matched population.

	PORTICO	ACURATE Neo	*p*-Value
	(*n* = 344)	(*n* = 344)	
Device failure ^1^	26 (7.6%)	25 (7.3%)	1.000
Procedural related death	1 (0.5%)	4 (2.0%)	0.372
Significant paravalvular leakage (>Grade II)	12 (3.5%)	16 (4.8%)	0.546
Elevated gradient (>20 mmHg)	4 (1.2%)	5 (1.5%)	0.752
Multiple valves	10 (2.9%)	7 (2.0%)	0.623
Conversion to sternotomy	5 (1.5%)	2 (0.6%)	0.451

Values are the mean ± SD, *n* (%). ^1^ Prothesis mismatch, mean aortic gradient ≥20 mmHg or peak velocity ≥3 m/s, moderate or severe prosthetic valve aortic regurgitation of the first implanted valve and multiple events possible.

**Table 4:** Reasons for conversion to open heart surgery (entire population).

	PORTICO	ACURATE
	(*n* = 5)	(*n* = 7)
Reasons for conversion		
Coronary impairment	0 (0.0%)	1 (14.3%)
Embolization	3 (60%)	2 (28.6%)
Pericardial effusion	1 (20%)	4 (57.1%)
Severe mitral regurgitation (due to wire)	1 (20%)	0 (0.0%)

Values are *n* (%).

## Supplementary Materials

The following supporting information can be downloaded at: https://www.mdpi.com/article/10.3390/jcm11144228/s1, Table S1: Prosthesis parameters, sizing recommendations and sheath profiles for ACURATE neo and PORTICO; Table S2: Procedural characteristics and in-hospital complications of patients treated with PORTICO and of the entire and the matched population treated with ACURATE neo; Table S3: Device failure of patients treated with PORTICO and ACURATE neo of the entire population; Table S4 Reasons for Conversion (Entire population).
