# Supplementary material for: Multi-Center Comparison of Two Self-Expanding Transcatheter Heart Valves: A Propensity Matched Analysis
Source: J Clin Med. 2022 Jul 21;11(14):4228. doi: 10.3390/jcm11144228 (PMC9318122; doi:10.3390/jcm11144228)
Supplement: Supplementary file 1 [file jcm-11-04228-s001.zip › jcm-1779513-supplementary.pdf]

Supplemental Table S1: Prosthesis parameters, sizing recommendations and sheath profiles for ACURATE neo and PORTICO

	ACURATE neo			PORTICO			
**Prosthesis parameters**							
Sizes (mm)	S = 23	M = 25	L = 27	23	25	27	29
Outer diameter (mm)	23	25	27	23	25	27	29
Minimum vessel diameter (mm)	≥ 6.0mm (18 F)	≥ 6.0mm (18 F)	≥ 6.0mm (18 F)	≥ 6.0mm (18 F)	≥ 6.0mm (18 F)	≥ 6.5mm (19 F)	≥ 6.5mm (19 F)
**Recommended annulus ranges**							
Area (cm^2)	3.46-4.15	4.15-4.91	4.91-5.73	2.77–3.46	3.38–4.15	4.05–4.91	4.79–5.73
Perimeter (mm)	66-72	72-79	79-85	60-66	66-73	72-79	79-85
Diameter (mm)	21.1-22.9	22.9-25.0	25.0-26.9	18.9-22.1	22.1-23.1	22.9-25.0	25.0-26.9

Supplemental Table S2: Procedural characteristics and in-hospital complications of patients treated with PORTICO and of the entire and the matched population treated with ACURATE neo

		Entire Population	
	PORTICO *(n = 344)*	ACURATE neo *(n = 1247)*	p Value
**Procedural data**			
Conscious sedation	308 (89.5%)	1110 (89.0%)	0.859
THV Size			<0.001
23	6 (1.7%)	277 (22.2%)	
25	93 (27.0%)	531 (42.6%)	
27	123 (35.8%)	438 (35.2%)	
29	122 (35.5%)	0 (0.0%)	
Predilatation	275 (79.9%)	876 (70.3%)	0.001
Postdilatation	64 (18.6%)	384 (30.8%)	<0.001
Cerebral protection	2 (0.6%)	28 (2.2%)	0.074
Procedural time (min)	52.0 (40.0–69.0)	50.0 (34.0–67.0)	0.004
Contrast (ml)	120.0 (100.0–160.0)	100.0 (80.0–120.0)	<0.001
Fluoroscopy dose (Gy)	1466.5 (29.1–3463.8)	133.1 (18.8–2767.5)	<0.001
Fluoroscopy time (s)	12.5 (9.2–17.1)	9.7 (7.3–13.6)	<0.001

	Entire Population		
	PORTICO	ACURATE neo	p Value
	(n = 344)	*(n = 1247)*	
**Echocardiographic characteristics**			
Postprocedural mean gradient (mmHg)	8.0 (6.0–10.0)	8.0 (6.0–11.0)	0.443
Postprocedural max gradient (mmHg)	13.0 (10.0–18.0)	14.0 (10.8–19.2)	0.425
**Clinical events**			
Major stroke /minor stroke/ TIA	13 (3.8%)	35 (2.8%)	0.446
Major vascular complications	9 (4.5%)	42 (6.8%)	0.334
Life-threatning bleeding (VARC)	2 (1.0%)	7 (1.1%)	1.000
Renal failure (AKIN 2/3)	12 (3.5%)	35 (2.8%)	0.636
Coronary artery obstruction with PCI	0 (0.0%)	7 (1.1%)	0.205
Myocardial infarction	6 (3.0%)	22 (3.5%)	0.898
Permanent pacemaker implantation [1]	52 (18.7%)	126 (11.5%)	0.002
Days in hospital	7.0 (6.0–10.0)	8.0 (6.0–10.0)	0.817
Days on intensive care unit	2.0 (1.0–3.0)	2.0 (1.0–3.0)	0.016
In-hospital mortality	10 (2.9%)	22 (1.8%)	0.264

Values are mean SD, n (%), or median (interquartile range). [1] Excluding patients with pacemaker at baseline (n = 213 in unmatched population).

	Entire Population		
	PORTICO<br>*(n = 344)*	ACURATE neo<br>*(n = 1247)*	p Value

VARC = Valve Academic Research Consortium, AKIN = Acute Kidney Injury Network, TIA = transitory ischemic attack.

Supplemental Table S3: Device failure of patients treated with PORTICO and ACURATE neo of the entire population

	PORTICO *(n = 344)*	ACURATE neo *(n = 1247)*	p Value
Device failure [1]	26 (7.6%)	88 (7.1%)	0.841
Procedural related death	1 (0.5%)	6 (1.0%)	1.000
Significant PVL (> Grade II)	12 (3.5%)	42 (3.4%)	1.000
Elevated gradient (>20mmHg)	4 (1.2%)	24 (2.0%)	0.475
Multiple valves	10 (2.9%)	19 (1.5%)	0.141
Conversion to sternotomy	5 (1.5%)	7 (0.6%)	0.148

Values are mean SD, n (%).

[1] Prothesis mismatch, mean aortic gradient > 20 mmHg or peak velocity > 3 m/s, moderate or severe prosthetic valve aortic regurgitation of the first implanted valve, multiple events possible.

PVL = paravalvular leakage

Table S4: Reasons for Conversion (Entire population)

	PORTICO<br>*(n = 5)*	ACURATE neo<br>*(n = 7)*	p Value
Reasons for conversion			
Coronary impairment	0 (0.0%)	1 (14.3%)	
Embolisation	3 (60%)	2 (28.6%)	
Pericardial effusion	1 (20%)	4 (57.1%)	
Severe mitral regurgitation (due to wire)	1 (20%)	0 (0.0%)	

Values are n (%).
